# Metabolomic profile of cancer stem cell‐derived exosomes from patients with malignant melanoma

**DOI:** 10.1002/1878-0261.12823

**Published:** 2020-11-25

**Authors:** José Luis Palacios‐Ferrer, María Belén García‐Ortega, María Gallardo‐Gómez, María Ángel García, Caridad Díaz, Houria Boulaiz, Javier Valdivia, José Miguel Jurado, Francisco M. Almazan‐Fernandez, Salvador Arias‐Santiago, Víctor Amezcua, Héctor Peinado, Francisca Vicente, José Pérez del Palacio, Juan A. Marchal

**Affiliations:** ^1^ Biopathology and Regenerative Medicine Institute (IBIMER) Centre for Biomedical Research (CIBM) University of Granada Spain; ^2^ Department of Human Anatomy and Embryology Faculty of Medicine University of Granada Spain; ^3^ Instituto de Investigación Biosanitaria de Granada (ibs.GRANADA) Spain; ^4^ Excellence Research Unit ‘Modeling Nature’ (MNat) University of Granada Spain; ^5^ Department of Oncology Virgen de las Nieves University Hospital Granada Spain; ^6^ Department of Biochemistry, Genetics and Immunology Singular Research Centre of Galicia (CINBIO) University of Vigo Spain; ^7^ Department of Biochemistry 3 and Immunology Faculty of Medicine University of Granada Spain; ^8^ Fundación MEDINA Centro de Excelencia en Investigación de Medicamentos Innovadores en Andalucía Parque Tecnológico Ciencias de la Salud Granada Spain; ^9^ Department of Oncology San Cecilio University Hospital Granada Spain; ^10^ Department of Dermatology San Cecilio University Hospital Granada Spain; ^11^ Department of Dermatology Virgen de las Nieves University Hospital Granada Spain; ^12^ Department of Medicine Faculty of Medicine University of Granada Spain; ^13^ Microenvironment and Metastasis Laboratory Molecular Oncology Program Spanish National Cancer Research Centre (CNIO) Madrid Spain

**Keywords:** biomarkers, cancer stem cells, exosomes, malignant melanoma, metabolomics

## Abstract

Malignant melanoma (MM) is the most aggressive and life‐threatening form of skin cancer. It is characterized by an extraordinary metastasis capacity and chemotherapy resistance, mainly due to melanoma cancer stem cells (CSCs). To date, there are no suitable clinical diagnostic, prognostic or predictive biomarkers for this neoplasia. Therefore, there is an urgent need for new MM biomarkers that enable early diagnosis and effective disease monitoring. Exosomes represent a novel source of biomarkers since they can be easily isolated from different body fluids. In this work, a primary patient‐derived MM cell line enriched in CSCs was characterized by assessing the expression of specific markers and their stem‐like properties. Exosomes derived from CSCs and serums from patients with MM were characterized, and their metabolomic profile was analysed by high‐resolution mass spectrometry (HRMS) following an untargeted approach and applying univariate and multivariate statistical analyses. The aim of this study was to search potential biomarkers for the diagnosis of this disease. Our results showed significant metabolomic differences in exosomes derived from MM CSCs compared with those from differentiated tumour cells and also in serum‐derived exosomes from patients with MM compared to those from healthy controls. Interestingly, we identified similarities between structural lipids differentially expressed in CSC‐derived exosomes and those derived from patients with MM such as the glycerophosphocholine PC 16:0/0:0. To our knowledge, this is the first metabolomic‐based study aimed at characterizing exosomes derived from melanoma CSCs and patients' serum in order to identify potential biomarkers for MM diagnosis. We conclude that metabolomic characterization of CSC‐derived exosomes sets an open door to the discovery of clinically useful biomarkers in this neoplasia.

## Graphical abstract

Malignant melanoma (MM) is the most aggressive form of skin cancer, with increasing incidence worldwide. To date, there are no suitable clinical diagnostic, prognostic or predictive biomarkers for MM. Our data highlight the potential of metabolomic characterization of cancer stem cell‐ or serum‐derived exosomes using high‐resolution mass spectrometry for the discovery of clinically useful MM biomarkers.

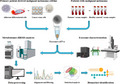

**Abbreviations**
CSCs: cancer stem cells
EVs: extracellular vesicles
HCs: healthy controls
HPLC: high‐performance liquid chromatography
HRMS: high‐resolution mass spectrometry
MM: malignant melanoma
MMPs: malignant melanoma patients
PCA: principal component analysis
PLS‐DA: partial least squares discriminant analysis
Q‐TOF‐MS: quadrupole time‐of‐flight mass spectrometer
ROC: receiver operating characteristic curve
TIC: total ion chromatograms
TME: tumour microenvironment
VIP: variable importance in projection

## 1 Introduction

Malignant melanoma (MM) is a highly aggressive form of skin cancer whose incidence continues increasing worldwide at a great rate. It is known that this aggressiveness is mainly due to intratumoral heterogeneity. In fact, tumour cells are hierarchically organized and sustained by a subpopulation of cells, known as cancer stem cells (CSCs, or tumour‐initiating cells), which possess stem‐like functional properties such as self‐renewal ability and multipotency [1, 2]. They are responsible for tumour initiation, maintenance, progression, metastasis and recurrence. In addition, CSCs are remarkably resistant to radiotherapy and chemotherapy as a consequence of their particular biology. Moreover, the CSC phenotype is not a rigid state and the intratumoral heterogeneity of cancer also extends to CSC characteristics, mainly due to tumour microenvironment (TME) [3]. In particular, malignant melanoma CSCs can be identified by the expression of specific markers and functional assays, and their stem‐like properties have been demonstrated *in vitro* and *in vivo* [4, 5].

Although significant efforts have been made in the last years, identification of useful diagnostic, prognostic and predictive biomarkers in MM remains challenging. Several candidate biomarkers have been proposed, but few have reached clinical application [6]. Thus, it is important to discover specific useful biomarkers and develop methods that can sensitively detect this neoplasia at subclinical metastatic stages.

Recent investigations confirm that extracellular vesicles (EVs) and exosomes play a major role in cancer development. They have been recently defined as smaller EVs (sEVs) as a general term, according to minimal information for studies of extracellular vesicles (‘MISEV’) guidelines [7] proposed by the International Society for Extracellular Vesicles (ISEV). By transferring their cargo to target cells of different lineage, cancer cell‐derived exosomes are able to induce pathways involved in cancer initiation, sustenance, progression and metastasis [8]. They support tumour progression by promoting angiogenesis, immune system modulation and tumour parenchyma remodelling [9, 10]. In addition, EVs are also released by CSCs, influencing their surrounding niche. Indeed, CSC‐EVs can regulate direct crosstalk with other neoplastic cells or can modify normal surrounding cells to promote immune tumour escape, tumour growth and metastasis. Several studies have demonstrated that CSC‐derived EVs play a key role in tumour progression [11].

Exosomes have been involved in the metastatic organotropism mediated by the integrins present in their surface, and therefore, exosomal integrins could be used to predict organ‐specific metastasis [12]. Malignant melanoma‐derived exosomes are involved in the metastatic dissemination to regional lymph nodes [13] and distant organs by promoting the generation of premetastatic niches [14]. Melanoma‐derived exosomes contribute to metastatic invasion by carrying messenger proteins (e.g. the oncoprotein c‐MET) educating bone marrow‐derived cells towards a pro‐metastatic phenotype or influencing the behaviour of immune cells [15].

Importantly, the release of exosomes and other EVs into the different body biofluids allows their detection, being a major source of secreted biomarkers in circulation [12, 16, 17, 18]. Since cancer cells exhibit enhanced production of exosomes, their concentrations are increased in body fluids of cancer patients compared with healthy controls [8]. Thus, exosomes (and other EVs) could represent a rich source of noninvasive biomarkers for the diagnosis and prognosis of cancers, including MM, as well as therapeutic targets [17, 19, 20].

In this context, metabolomics as emerging ‘omic’ research technology represents a potential tool for biomarkers' discovery. Metabolomics refers to the systematic identification and quantification of the complete set of low molecular weight metabolites, known as metabolome, which are context‐dependent and vary according to the physiology, developmental or pathological state of a cell, tissue or organism [21]. The study of the complete metabolome is technically challenging, due to its diversity, and multiple strategies are employed to provide a broad metabolic coverage. In this regard, mass spectrometry (MS) is the gold standard analytical platform for metabolomics, as it provides high sensitivity, versatility and reproducibility [18, 21, 22]. In order to extract useful biological information from large and complex data sets generated by mass spectrometers, univariate (*t*‐test, ANOVA) and multivariate (PCA, PLS‐DA) statistical analyses are used [18, 23].

Over the last decade, several studies using omic technologies have enhanced the development and validation of biomarkers currently used in the diagnosis, prognosis and treatment response prediction in MM [24], but unfortunately none of the metabolites identified as potential biomarkers has been proven to be clinically useful so far. Some metabolomic studies have evaluated metabolic changes in melanoma using various analytical techniques in both *in vivo* and *in vitro* models, but mainly using gas chromatography–mass spectrometry (GC‐MS) [25] or nuclear magnetic resonance (NMR) [26], not many of them have been carried out using liquid chromatography–mass spectrometry (LC‐MS) [27].

Here, we have characterized a patient‐derived MM cell population enriched in CSCs and have analysed the metabolomic profile of exosomes derived from these MM cells and from serum of patients with MM using a high‐resolution mass spectrometry untargeted approach. To our knowledge, we report for the first‐time differences in exosome metabolomic profile from CSC‐enriched melanospheres versus MM‐differentiated cells. We also report metabolomic differences between serum‐derived exosomes from patients with MM at several stages of the disease compared with those derived from healthy controls.

## 2 Materials and methods

### 2.1 Cell culture and CSC enrichment

The human primary Mel1 MM cell line comes from a malignant metastatic melanoma (stage M1a) skin biopsy (BBSPA‐Mel#1) and was provided by the Biobank of the Andalusian Public Health System (Spain). This cell line is hypotriploid (complex karyotype with multiple numerical and structural chromosome abnormalities), MelA‐positive, p53‐positive and S100‐positive, and has high tumorigenic ability. Mel1 adherent cells were maintained in standard culture conditions. Enriched Mel1 CSC subpopulations were obtained after culturing as primary and secondary spheroids in serum‐free medium and in anchorage‐independent conditions [28]. Mel1 adherent cells were cultured in a humid incubator at 37 °C and 5% CO_2_, with DMEM (Dulbecco's modified Eagle's medium) (Sigma‐Aldrich, St. Louis, MO, USA) supplemented with 10% heat‐inactivated fetal bovine serum (FBS) (Gibco, Grand Island, NY, USA) and 1% penicillin/streptomycin (P/S) (Sigma‐Aldrich) in 75‐cm^2^ flask culture (Nunc, Roskilde, Denmark), unless otherwise indicated. FBS was inactivated by heating at 56 °C for 45 min. Cells were assayed for mycoplasma contamination. Enriched Mel1 CSC subpopulations were obtained as follows: for primary spheroids, culture cells were collected by centrifugation (352 ***g*** for 10 min) and the pellet was resuspended twice in PBS (phosphate‐buffered saline). Then, resuspended cells were plated in serum‐free sphere culture medium (DMEM:F12, 1% P/S, B27, 10 µg·mL^−1^ ITS, 1 µg·mL^−1^ hydrocortisone, 4 ng·mL^−1^ heparin, 20 ng·mL^−1^ EGF, 10 ng·mL^−1^ FGF, 10 ng·mL^−1^ IL6, 10 ng·mL^−1^ HGF) in ultra‐low adherence 6‐well plates (Corning, Corning, NY, USA) for 72 h. For the secondary sphere culture, cells from primary spheroids were collected by centrifugation (1500 r.p.m. for 10 min), and then, the pellet was resuspended in DMEM F12 sphere medium mechanically disrupted with a pipette and by syringing three to five times through a sterile 25‐gauge needle. After that, cells were plated, resuspended and incubated for 72 h in sphere culture medium in ultra‐low adherence 6‐well plates.

### 2.2 Sphere‐forming assay

To determine the self‐renewal ability of the Mel1 CSC population, a sphere‐forming assay was performed [29]. Mel1 cell lines were grown as spheroids as described above: 2.5 × 10^5^ cells were washed with PBS and resuspended in sphere culture medium in ultra‐low adherence 6‐well plates (Corning). Spheres > 75 µm diameter were counted after 3 days by light microscopy. For the secondary sphere‐forming assay, 2.5 × 10^5^ single cells derived from primary spheroids were plated and resuspended in sphere culture medium in ultra‐low adherence 6‐well plates. Diameters were measured using the imagej software.

### 2.3 Colony‐formation assay

The clonogenic capability of Mel1 CSCs was determined by a colony‐formation assay in soft agar as previously described [29] with minor modifications. Briefly, 10^4^ cells coming from secondary spheroids were seeded in 0.4% cell agar base layer, which was on top of 0.8% base agar layer in 6‐well culture plates. Then, cells were incubated for 23 days at 37 °C and 5% CO_2_, adding 100 µL of DMEM (10% FBS, 1% P/S) every 1–2 days. Cell colony formation was then examined under a light microscope after staining with 0.1% iodonitrotetrazolium chloride (Sigma‐Aldrich). The size of colonies was measured using imagej™ software (Rasband, W.S., ImageJ, U. S. National Institutes of Health, Bethesda, MD, USA).

### 2.4 ALDEFLOUR assay and phenotypic characterization by flow cytometry

The analysis of CD20 and CD44 surface markers and the ALDH1 activity were performed using a Becton Dickinson FACSCanto II Flow Cytometer from the CIC Scientific Instrumental Centre (University of Granada) as previously described [29]. Briefly, ALDEFLUOR assays (Stem Cell Technologies, Vancouver, BC, Canada) to detect ALDH1 activity in viable cells were performed according to the manufacturer's instructions. Cells were suspended in ALDEFLUOR assay buffer containing ALDH1 substrate (BAAA, 1 μmol·L^−1^ per 1 × 10^6^ cells) and incubated for 45 min at 37 °C in darkness. Diethylaminobenzaldehyde (DEAB) was used as an ALDH1 inhibitor to set ALDH1 gates. The bright fluorescent ALDH1‐expressing cells were detected in the green fluorescent channel (520–540 nm). Cell surface levels of CD44 and CD20 were determined with anti‐human antibodies CD44‐phycoerythrin (PE) and CD20‐allophycocyanin (APC) (Miltenyi Biotec, Bergisch Gladbach, Germany), respectively. The bright fluorescent PE and APC were detected in red (564–606 nm) and blue (650–670 nm), respectively. All samples were analysed on a FACSCanto II (BD Biosciences, San Jose, CA, USA) using the facsdiva software.

### 2.5 Side population assays

Hoechst 33342 exclusion (side population) assays were carried out as previously described [30] to analyse cells overexpressing ABC transporters. Single‐cell suspension obtained from parental cell lines and melanospheres were stained with Hoechst 33342 (Sigma‐Aldrich) dye. As negative controls, verapamil (Sigma‐Aldrich) was used for maintaining the efflux channel closed, inhibiting the capacity to efflux Hoechst 33342 by cells. The bright fluorescent cells were measured by flow cytometry in Hoechst blue (440/40) and Hoechst red (695/40) of a FACScan Aria III (BD Biosciences) using FACSDIVA software from the CIC Scientific Instrumental Centre (University of Granada). Cells with the ability to efflux Hoechst 33342 were considered as the side population (SP).

### 2.6 Serum sample collection and preparation

Serum samples were obtained from the Oncology Service at the University Hospital Virgen de las Nieves of Granada and University Hospital San Cecilio of Granada (Spain). The ethics committee from both hospitals approved the study (number: 32140085), and all clinical investigations were conducted according to the principles expressed in the Declaration of Helsinki (‘Ethical Principles for Medical Research Involving Human Subjects’). Written informed consent was obtained from all patients and controls before their enrolment in the study. Samples were obtained from serum of patients with MM (MMPs) (*n* = 20) and healthy controls (HCs) (*n* = 14). MMPs presented different stages of the disease, namely stage I (*n* = 5), stage II (*n* = 5), stage III (*n* = 5) and stage IV (*n* = 5). Samples were collected in BD vacutainer SSTII advanced tubes (Becton Dickinson, Franklin Lakes, NJ, USA) with silica to activate clotting of the specimen, incubated at room temperature for 30 min and centrifuged for 10 min at 1400 ***g***. Afterwards, the supernatant (around 1 mL) was carefully aspirated and stored at −80 °C until the examination.

### 2.7 Exosome isolation and purification

For exosome isolation, Mel1 cells were cultured in 75‐cm^2^ flasks culture in standard anchorage‐dependent culture conditions with DMEM supplemented with 1% P/S and 10% heat‐inactivated exosome‐depleted FBS, until 80% confluence. FBS was depleted of bovine exosomes by ultracentrifugation at 100 000 ***g*** for 70 min [12]. Exosomes were also isolated from Mel1 CSCs: a total of 3 × 10^6^ cells were cultured as primary and, then, as secondary spheroids in anchorage‐independent and serum‐free conditions, as described above. Supernatant fractions collected from cell cultures after 72 h were centrifuged at 500 ***g*** for 10 min to remove any cell contamination and debris. Exosomes from cell‐free culture supernatants were purified by sequential centrifugation as previously described by Costa‐Silva *et al*. [31] with minor modifications. Briefly, to remove any possible apoptotic bodies, dead cells and large cell debris, the supernatants were first spun at 10 000 ***g*** for 40 min. Exosomes were collected by ultracentrifugation at 100 000 ***g*** for 80 min. Exosome pellets were washed in 35 mL PBS and pelleted again by ultracentrifugation at 100 000 ***g*** for 80 min (Beckman SW28 Rotor). In addition, serum‐derived exosomes from MMPs and HCs were isolated following the same protocol described above, but washing the exosome pellets in 10 mL PBS. The final pellet was resuspended in 100 μL of PBS and stored frozen at −80 °C for further analyses. Repeated freezing and thawing of the exosome suspensions were avoided.

### 2.8 Transmission and scanning electron microscopy

Transmission electron microscopy (TEM) and scanning electron microscopy (SEM) analyses were performed at the Centro de Instrumentación Científica (CIC, University of Granada). For TEM and SEM, samples were negatively stained with uranyl acetate as follows: a 30 μL drop of the exosome sample was placed on a carbon‐coated 300‐mesh grid and allowed to adsorb at room temperature for 5 min. The grids were then washed in drops of ultrapure water for 1 min. Adsorbed exosomes were negatively stained by placing the grids on a drop of 1% uranyl acetate in aqueous suspension for 1 min. The excess fluid was slightly drained with filter paper, and then, sample grids were dried at room temperature for 6 min. The preparations were examined with a LIBRA 120 PLUS transmission electron microscope (Carl Zeiss SMT, Oberkochen, Germany) at an acceleration voltage of 120 kV, and the HITACHI, S‐510 scanning electron microscope. Then, the samples were determinate with the Edwin‐Róntec microanalysis system.

In addition, pellets obtained from CSC cultures were immersed in 4% paraformaldehyde and 0.1 m PBS for 4 h at 4 °C and washed in sucrose in 0.1 m PBS overnight. The fractions were incubated by increased alcohol concentrations and were cut in semi‐thin sections at 0.5 µm with tissue processor (TP1020; Leica, Wetzlar, Germany).

### 2.9 Atomic force microscopy

Atomic force microscopy (AFM) analyses were performed at the Centro de Instrumentación Científica (CIC, University of Granada). For AFM, purified exosomes were diluted 1 : 10 in deionized water. A 10 μL drop of exosome suspension was adsorbed to freshly cleaved mica sheets at room temperature for 10 min and rinsed with deionized water to remove salt precipitates. The sheets were then completely dried under a gentle stream of argon gas (Ar). The preparations were examined with an NX20 Atomic Force Microscope (Park Systems, Suwon, South Korea), and images were visualized and processed using the park systems xei software (Park Systems, Suwon, South Korea). Measurements were carried out with ACTA cantilevers (40 N·m^−1^) and in noncontact mode.

### 2.10 Western blot analysis

Exosome pellets were isolated from 100 mL of cell culture supernatants of adherent cells and CSCs and from 600 µL of patients' serum. The final pellets were resuspended in 100 μL of PBS and stored at 4 °C for further protein quantification. The protein concentrations were measured using the BCA Protein Assay Kit (Pierce, Rockford, IL, USA) according to the manufacturer's instructions. Proteins extracts (30 μg) were denatured at 95 °C for 5 min in loading buffer (containing Tris – pH 6.8, SDS, glycerol, β‐mercaptoethanol and bromophenol blue). Proteins were subjected to 4–20% Mini‐PROTEAN TGX (Bio‐Rad, Hercules, CA, USA) gel together with Precision Plus Protein™ Kaleidoscope Prestained Protein Standards (Bio‐Rad). The samples were transferred to a nitrocellulose membrane (Trans‐Blot, Mini Format, Bio‐Rad) using a transfer apparatus according to the manufacturer's protocols (standard programme: 25 V for 30 min) (Bio‐Rad). After incubation with 5% skimmed milk in PBS‐Tween 0.1% for 1 h at room temperature, the membranes were incubated overnight with antibodies against CD9 (dilution 1/1500; eBioscience, San Diego, CA, USA), CD63 (dilution 1/500; Santa Cruz Biotechnology, Dallas, TX, USA), CD271 (dilution 1/500; Abcam, Cambridge, UK) and Alix (dilution 1/1000; Cell Signaling, Danvers, MA, USA). Membranes were then incubated with conjugated goat anti‐mouse secondary antibody and goat anti‐rabbit secondary antibody for 2 h, and signals were detected using the ECL‐PLUS y ECL PRIME (Amersham Biosciences, Little Chalfont, UK). The bands were visualized with medical photographic films (AGFA, Mortsel, Belgium).

### 2.11 Exosome size analysis

Analyses were performed on NanoSight NS500 Instruments (Malvern Instruments, Malvern, UK). The instrument was equipped with a 488‐nm laser, a high‐sensitivity CMOS camera and a syringe pump. Exosomes were diluted 1 : 1000 in PBS buffer to obtain a concentration range (1–10 × 10^8^ particles·mL^−1^). The measurements were analysed using the nta2.3 software (Malvern) after filming three 60‐s videos.

### 2.12 Immunogold labelling by transmission electron microscopy

Immunogold labelling of exosomes was carried out at the Andalusian Centre for Nanomedicine (Bionand, Málaga, Spain). Exosome suspensions were put on copper grid with formvar–carbon and incubated for 15 min at RT. They were dried slightly and diluted in 15 µL of 2% paraformaldehyde in 0.1 m PBS and incubated for 10 min. The samples were transferred to a 15 µL drop of 2% BSA in 0.1 m PBS, plus the primary antibody anti‐human CD63 clone H5C6 (RUO) (Becton Dickinson) diluted 1/500 and incubated for 1.5 h at room temperature within a humid chamber. After several PBS washes, the grid was incubated with the anti‐mouse IgG (whole molecule)‐gold 10 nm secondary antibody (Sigma‐Aldrich) and incubated 1 h at room temperature within a humid chamber. The samples were marked with a negative stain by using 15 μL of 1% uranyl acetate in Milli‐Q water for 15 s. The preparations were examined with a LIBRA 120 PLUS transmission electron microscope (Carl Zeiss SMT, Oberkochen, Germany).

### 2.13 LC‐HRMS analysis of exosomes

The metabolomic analyses of exosomes isolated from cell culture supernatant and patients' serum samples were performed in Fundación MEDINA (Centro de Excelencia en Investigación de Medicamentos Innovadores en Andalucía) as described by García‐Fontana *et al*. [32] with minor modifications. Exosome samples were kept at 4 °C during the analytical process. Sample preparation for LC‐HRMS analysis was performed as follows. Exosome samples were thawed on ice and vortexed. Proteins were removed from exosome suspension using methanol (1 : 3 exosomes : methanol), shaken (60 s), sonicated (1 min) and shaken again. Samples were then centrifuged at 22 600 ***g*** for 15 min at 4 °C. Supernatants were collected and dried under an N_2_ air stream. Dried samples were reconstituted in 90 μL of mobile phase (50% H_2_O and 50% acetonitrile at 0.1% of formic acid) and transferred to the analytical vials. Samples were stored at 4 °C and analysed in quadruplicate within 24 h of reconstitution using AB SCIEX TripleTOF 5600 quadrupole time‐of‐flight mass spectrometer (Q‐TOF‐MS) (AB SCIEX, Concord, ON, Canada) coupled to an high‐performance liquid chromatography (HPLC) system, in positive electrospray ionization (ESI) mode.

Before HRMS analysis, chromatographic separation was performed by Agilent Series 1290 LC system (Agilent Technologies, Santa Clara, CA, USA), equipped with a reverse‐phase Atlantis T3 HPLC C18 column (C18: 2.1 mm × 150 mm, 3 mm) (Waters, Milford, MA, USA). For each sample, 5 μL was injected into the HPLC system. Samples were injected randomly in order to prevent any possible time‐dependent changes in the chromatographic profiling. Blank solvent (BS) and quality control (QC) samples were injected interspersed in the sequence run. The QC samples were prepared by pooling an equal volume of all exosome samples and injected every seven samples in order to check the stability and performance of the system. The BS samples were also run interspersed in the sequence to identify possible impurities of the solvents or extraction procedure and to check carryover contamination from intense analytes. Generic parameter settings for chromatographic separation and MS detection were used to obtain specific metabolomic fingerprints of the exosome preparations.

HRMS analysis was carried out using an information‐dependent acquisition (IDA) method to collect full‐scan MS and MS/MS information simultaneously. The method consisted of high‐resolution survey spectra from *m/z* 50 to *m/z* 1600, and the eight most intense ions were selected for acquiring MS/MS fragmentation spectra after each scan. An Automated Calibration Delivery System performed an exact mass calibration prior to each analysis.

#### 2.13.1 Data set creation

peakview software (AB SCIEX) was used in order to assess the analytical drift in terms of mass and retention time shift. markerview software (version 1.2.1.1; AB SCIEX) was used for processing the LC‐HRMS raw data. This software performs peak detection, alignment and data filtering, generating a feature table which defines measured *m/z*, retention time (RT) and integrated ion intensity. An automated algorithm in the RT range 0.8–19 min and *m/z* range 50–1600 was used for data mining. The intensity threshold of extraction was established at 100 counts per second. RT and *m/z* tolerances of 0.1 min and 15 p.p.m., respectively, were used for peak alignment. Background noise was removed by using a specific tool of markerview software. The analytical replicates of each sample were averaged.

#### 2.13.2 Analytical validation

Quality control distribution on PCA plot was used for analytical validation prior to the following analysis. After that, different approaches of data normalization (normalization by a QC reference sample, non‐normalized data, sum and median normalization), data transformation (logarithmic and cubic root transformation) and scaling (autoscaling and Pareto scaling) were evaluated in order to define which combination provides a better grouping of QC samples on PCA plot and a normal distribution of the data. Variables with unacceptable reproducibility (RSD > 30%) or detected in less than 50% of QC samples were also rejected from the data matrix.

#### 2.13.3 Data treatment

Statistical analyses were carried out using MetaboAnalyst 3.0 Web Server [23] as previously described [32]. Briefly, after data set creation, raw data were normalized (median normalization), transformed (cube root transformation) and scaled (Pareto scaling) in order to achieve a more Gaussian‐type distribution [33]. Then, filtering according to significant differences was done based on statistical analysis including both univariate (UVA) and multivariate (MVA). Statistical analyses were carried out to filter variables (metabolites) that are significantly different between the groups compared. UVA assesses the statistical significance of each peak/variable separately, while MVA takes into account the combination of the effects of multiple variables. For UVA, a double filtering procedure with *t*‐test (*P*‐value < 0.05) and fold change (FC > 1.5) was applied in order to identify differentially expressed mass signals between BS and exosome samples. This first filtering allowed removing background noise and preserving the peaks from true biological samples. Then, an ANOVA or *t*‐test‐based filtering (*P*‐value < 0.05) was used to detect differential metabolites between the sample groups, providing a quality criterion to assess variable relevance for further data analysis. For MVA, principal component analysis (PCA) and partial least squares regression (PLS‐DA) were carried out after ANOVA or *t*‐test filtering. PCA was applied to assess quality of the analytical system performance. PLS‐DA allowed discriminating variables that are responsible for variation between the comparison groups. For statistical validation, quality description by goodness of fit (*R* ^2^) and goodness of prediction (*Q* ^2^) was used. A powerful model for diagnostics should show high values of *R* ^2^ and *Q* ^2^ but also not vary more than 0.2–0.3. For metabolomic data, *R* ^2^ > 0.7 and *Q* ^2^ > 0.4 are considered acceptable values [33]. The models were also validated using 10‐fold cross‐validation. Receiver operator characteristic (ROC) curve analyses were carried out to evaluate the clinical utility of the metabolites selected as potential biomarkers [34]. The AUC provided in this work are flipped (1‐AUC), and consequently, they are always presented as being > 0.5 independently of the case–control ratios.

#### 2.13.4 Metabolite identification

In the next step, a stepwise search of the *m/z* and fragmentation spectrum of the possible candidates was performed on several online databases (METLIN, HMDB, LIPID MAPS, MassBank and PubChem), NIST MS/MS library (NIST14, version 2.2, National Institute of Standards and Technology 2014) and scientific literature. The mass tolerance was set at 10 ppm. For automatic MS and MS/MS elemental formula estimation, the Formula Finder plug‐in of peakview software (AB SCIEX) was used, setting the mass tolerance at 10 mDa. For those tentatively identified metabolites with a fragmentation spectrum available, the similarity between the experimental fragmentation spectrum obtained and the theoretical ones was checked using the fragmentation pane tool of peakview software for *in silico* fragmentation and also searching on the scientific literature. Only candidates whose presence in humans was likely were selected as possible novel MM biomarkers.

### 2.14 Statistical analysis

For Mel1 characterization, all data are presented as the mean ± standard deviation. Differences between groups were analysed for statistical significance using the two‐tailed Student's *t*‐test. *P*‐value of 0.05 was accepted as the statistical significance level.

## 3 Results

### 3.1 Characterization of primary Mel1 melanospheres

**Fig. 1:**
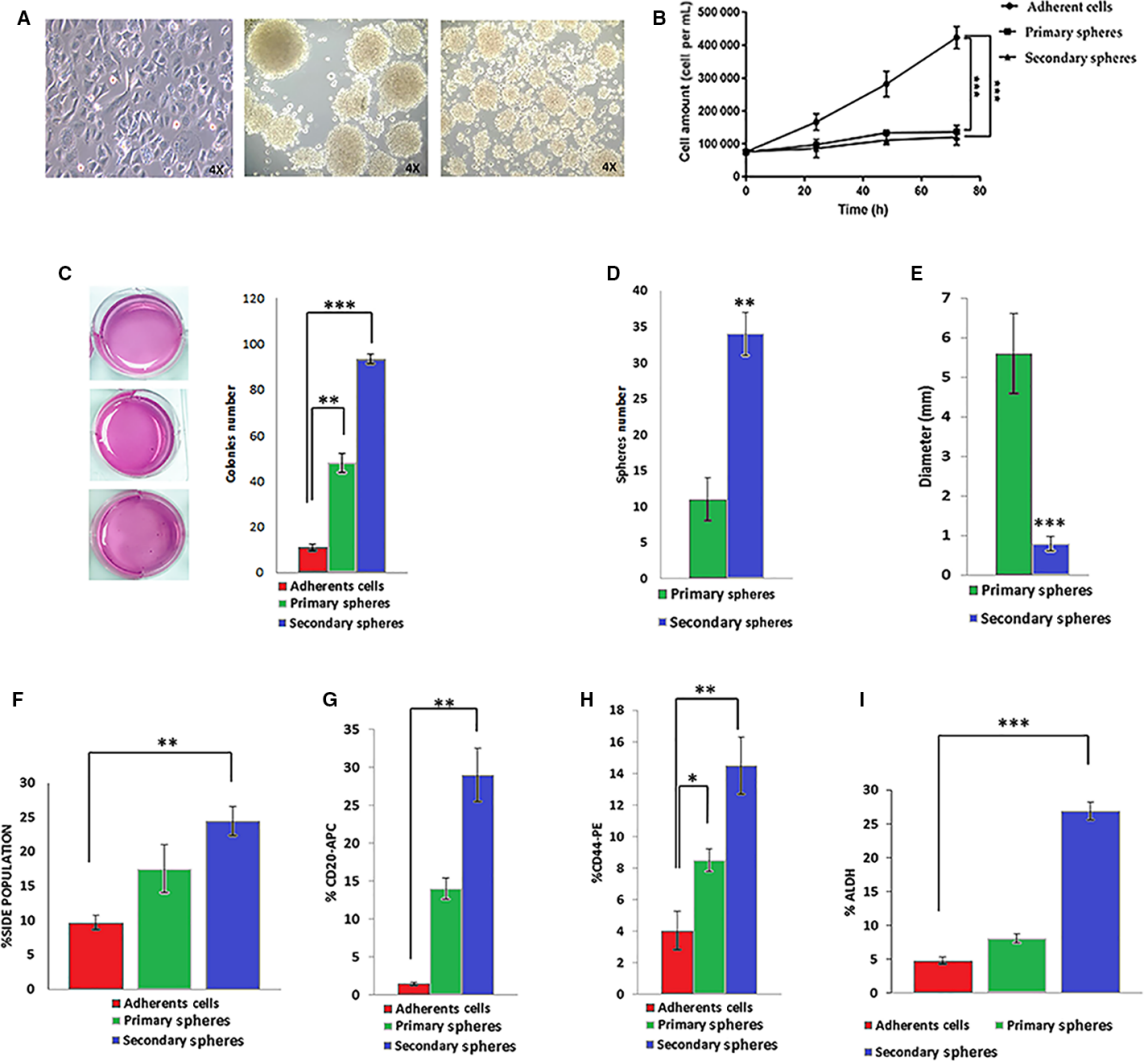
Characterization of Mel1 CSCs. (A) Representative light microscopy (4×) images of primary (left) and secondary (right) melanospheres formed from Mel1 cell line; (B) proliferation curves of Mel1 adherent cells and melanospheres cultured for 3 days and seeded with an equal number of cells at day 0; (C) representative optical image of the colonies formed by Mel1 cells coming from adherent cells, primary and secondary spheroids after 37 days soft agar culture in P6 well plates; stained with 0.1% iodonitrotetrazolium chloride; (D) number of primary and secondary spheres formed by Mel1 cell line growing in anchorage‐independent and serum‐free conditions. Spheres were counted after 3 days under light microscopy; (E) diameter of primary and secondary spheres, measured by imagej software; (F) side population determined in the different culture types; (G) percentage of CD20+ and (H) CD44+ in adherent cells and primary and secondary melanospheres, and (I) percentage of ALDH+ cells measured by flow cytometry. Data are graphed as mean ± SD from experiments carried out in triplicates (***P < 0.001; **P < 0.01; *P < 0.05).

For the enrichment of melanoma CSCs, we used a primary patient‐derived tumour cell line (Mel1) from an MM skin biopsy (stage M1a). We studied the anchorage‐independent growth of Mel1 spheres in serum‐free conditions [28] to determine their CSC characteristic phenotype by determining sphere‐forming ability, proliferation rate of primary and secondary melanospheres, clonogenic capacity by colony‐formation assay in soft agar, side population, CD20 and CD44 cell surface markers' expression and ALDH activity (Fig. 1).

Cells growing as melanospheres had a significantly lower proliferation rate when compared to the adherent cell culture of the same cell line, with a doubling time for adherent cells, and primary and secondary spheres of 31.6, 51.7 and 65.1 h, respectively (Fig. 1A,B). Therefore, melanospheres showed the slow‐cycling nature of stem cell populations. Under anchorage‐independent and serum‐free conditions Mel1 cells had the capacity of self‐renewal by the increased sphere number and size of melanospheres (Fig. 1A,C–E). Although a significantly higher number of secondary spheres were observed in comparison with primary spheres (Fig. 1A,D), the size was smaller in secondary spheres than in primary spheres, with average diameters of 5.6 and 0.8 mm, respectively (Fig. 1A,E). Moreover, Mel1 melanospheres showed a high capacity to form colonies in soft agar (Fig. 1C).

Accordingly, the rate of side population (SP) in melanospheres was significantly higher than in adherent cells (Fig. 1F). Thus, adherent cells displayed a 9.7% of SP, whereas primary and secondary Mel1 melanospheres showed 17.5% and 24.5%, respectively. Moreover, secondary spheres showed a significantly higher proportion of cells expressing both CD20+ and CD44+ markers with values of 29% and 14.5%, respectively, in comparison with primary melanospheres (CD44+: 14%, CD20+: 9%) and cells growing in adherent conditions, where only 1.5% and 5% were positive for CD20 and CD44, respectively (Fig. 1G,H). Regarding ALDH activity, both primary (8.1%) and secondary melanospheres (26.9%) displayed a significantly higher proportion of ALDH+ cells than the adherent ones (4.8%) (Fig. 1I).

Altogether, these results indicate that Mel1 cells growing as primary and secondary melanospheres in anchorage‐independent and serum‐free conditions constitute an enriched cell population with functional and phenotypic stemness properties. Since secondary melanospheres were most enriched in CSC properties, they were used for subsequent studies.

### 3.2 Isolation and characterization of exosomes derived from primary patient‐derived Mel1 CSCs and serum of patients with malignant melanoma

**Fig. 2:**
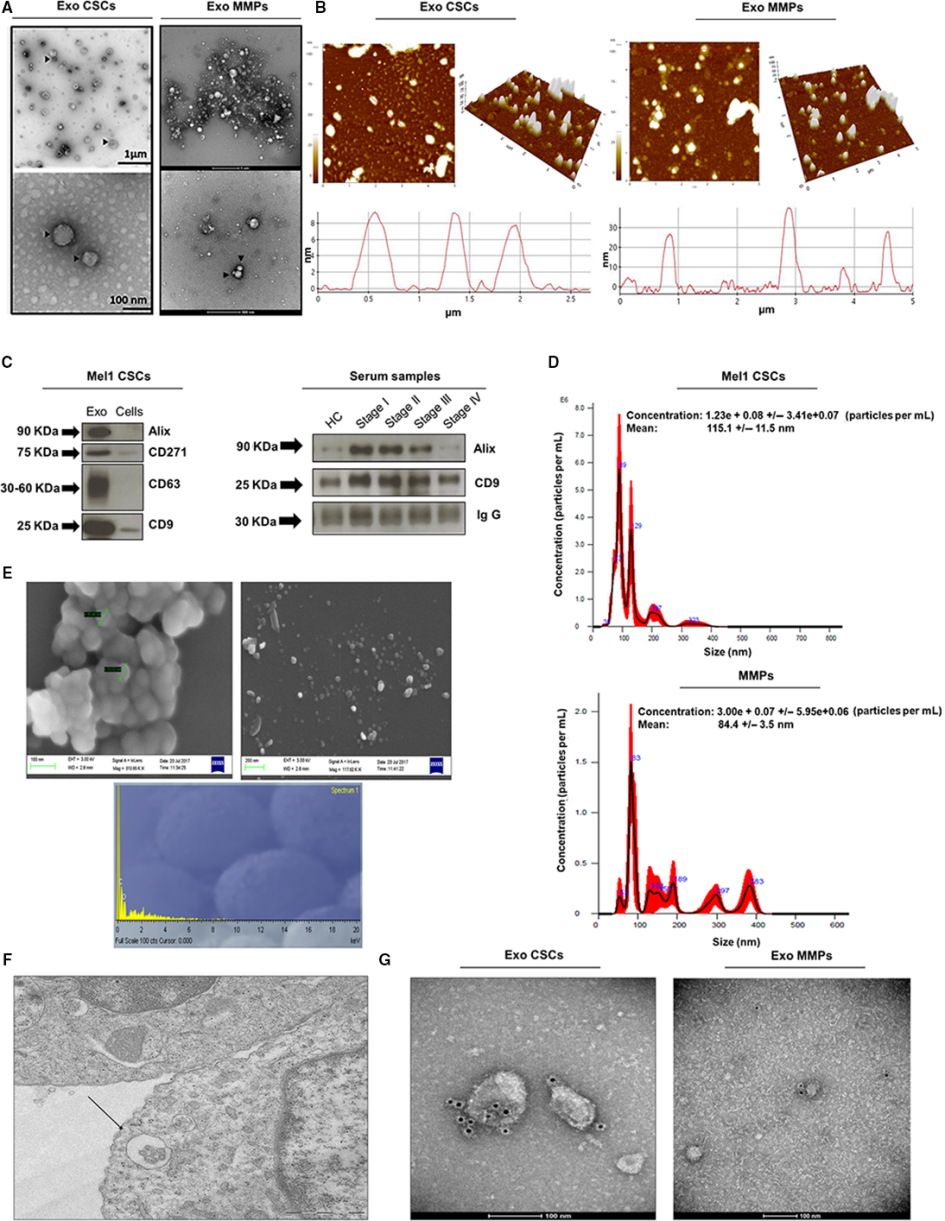
Characterization of exosomes derived from Mel1 secondary melanospheres and MMP serums. (A) Transmission electron microscopy images of isolated exosomes with a saucer‐like shape limited by a lipid bilayer. EVs isolated from Mel1 secondary melanospheres culture supernatants had diameters ranging from ~ 40 to 210 nm; those isolated from MMP in had a diameter ranging from ~ 30 to 140 nm. Images show exosomes derived from an MMP at stage IV. Black arrowheads point to exosomes; (B) topography of exosomes derived from Mel1 secondary melanospheres and MMP serum observed under atomic force microscopy (AFM). Exosomes on a mica surface revealed heterogeneity in size and shape as well as forming aggregates in both 2‐dimensional 2D (left) images and 3D profiles (right). Acquisition areas were 5 × 5 µm^2^; (C) western blot analysis of CD9, CD63, Alix exosomal surface markers and the CD271 melanoma stem cell marker in melanosphere‐derived exosomes and Mel1 CSCs. The expression of CD9 and Alix is also shown as representative exosomal surface markers in MMP serum‐derived exosomes. IgG was used as a positive control; (D) the size distribution of exosomes obtained from Mel1 CSC and MMP serum was analysed by NTA; (E) scanning electron microscopy images of CSC‐derived exosomes aggregated (left) and individualized (right) and microanalysis of particles (down) showing the particle composition; (F) multivesicular bodies observed by electron microscopy in Mel1 CSCs. Image obtained from paraffin sections; (G) immunogold using beads coated with an anti‐CD63 antibody in exosomes derived from Mel1 secondary melanospheres cultures (left) and from a stage IV MMP serum (right).

Based on their unique size and density, we isolated exosomes from the culture supernatant of Mel1 secondary spheres and MMP serum following the ultracentrifugation protocol described in the Materials and methods section. Exosome purification was confirmed by TEM, AFM, western blot, NanoSight and SEM (Fig. 2).

As shown in TEM images (Fig. 2A), vesicles obtained from Mel1 secondary melanospheres have a characteristic saucer‐like ultrastructure with diameters ranging from 40 to 210 nm and crescent‐shaped membrane invaginations limited by a lipid bilayer, while vesicles obtained from MMP serum had a diameter ranging from 30 to 140 nm (Fig. 2A). AFM images showed a heterogeneous organization of exosomes, in terms of the wide variation in shape and size as demonstrated in both 2‐dimensional (2D) images and topographic profiles, regardless of their origin (Fig. 2B). Western blot analysis showed that these extracellular Mel1 vesicles were positive to known exosome classic markers including CD63, Alix and CD9 (Fig. 2C). Moreover, we were able to detect the expression of the CD271 melanoma CSC marker in both exosomes and Mel1 secondary melanospheres. EVs isolated from serum of HCs and MMPs at different stages of the disease (stages I–IV) were also positive for Alix and CD9 markers (Fig. 2C).

Exosome size distribution determined by NTA Software confirmed the presence of particles with nanometric size in both types of samples, and the average concentration was 5.48 × 10^8^ particles·mL^−1^ for Mel1 CSC‐derived exosomes and 4.64 × 10^8^ particles·mL^−1^ for MMP serum‐derived exosomes. Mel1 CSC‐derived exosomes showed peaks around 115 nm corresponding to individual exosomes, while larger size peaks were related to exosome aggregates (Fig. 2D), which was also confirmed by SEM (Fig. 2E). The microanalysis determined that the majority component was carbon, which confirmed the organic origin of the samples (Fig. 2E).

Furthermore, we were able to detect multivesicular bodies with spheroid structures inside surrounded by a phospholipid bilayer in Mel1 secondary melanospheres (Fig. 2F). Finally, the morphology and size of exosomes were also verified by immunogold using beads coated with an anti CD63 antibody. Black punctate regions indicate a positive staining for CD63 around the exosome membranes from both Mel1 CSC‐derived exosomes and MMP serum‐derived exosomes (Fig. 2G). Exosomes derived from Mel1 differentiated adherent cells were also isolated and characterized according to the same criteria (Fig. S1).

### 3.3 LC‐HRMS metabolomic and chemometric analysis of primary patient Mel1‐derived exosomes

**Fig. 3:**
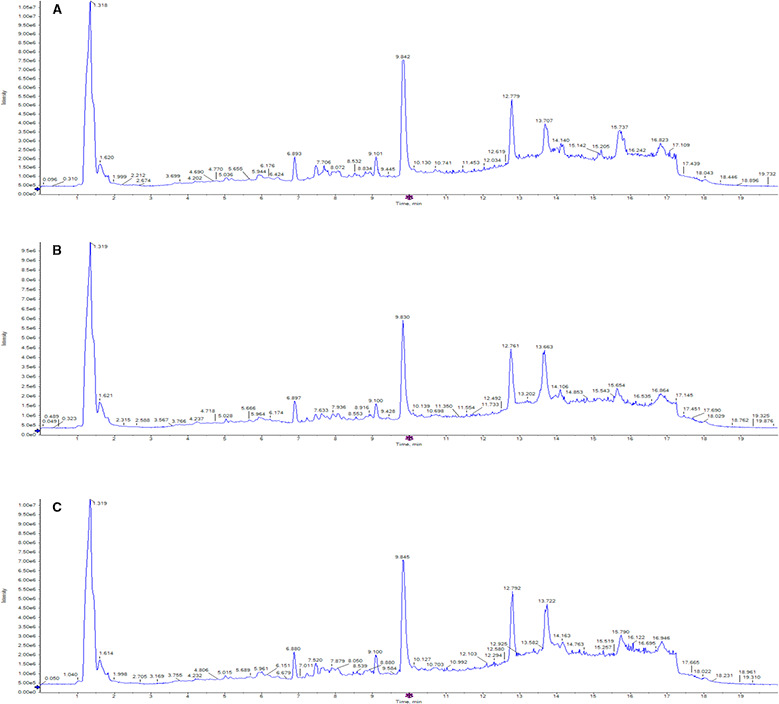
Representative HPLC/MS total ion chromatograms (TICs) of metabolites present in exosomes derived from Mel‐1 cell line. TIC corresponding to representative exosome samples derived from (A) adherent cells, (B) primary melanospheres and (C) secondary melanospheres, scanned by positive ion mode. The *x*‐axis represents the chromatographic retention time, while the *y*‐axis represents the intensity. Methanol was used for metabolite extraction.

Metabolomic characterization was first performed with exosomes isolated from adherent Mel1 tumour cells and from Mel1 CSCs. The HPLC‐Q‐TOF‐MS total ion chromatograms (TICs) observed in the positive ionization mode for the metabolites extracted from exosome samples showed excellent reproducibility in terms of retention time and signal intensity, suggesting a low analytical drift across the whole set of samples (Fig. 3). A positive ionization data matrix of 2486 mass signals was obtained as an outcome of the peak picking and alignment procedures. In order to filter the results and minimize the signal redundancy, only peaks representing monoisotopic ions (signals with the lowest *m/z* value within an isotope pattern) were selected (281 peaks) and subjected to the chemometric analysis. After that, raw data were normalized, transformed and scaled, and a first filtering by *t*‐test and twofold change was performed in order to discard mass signals present in blank solvent samples and, therefore, not exclusively present in biological samples.

**Fig. 4:**
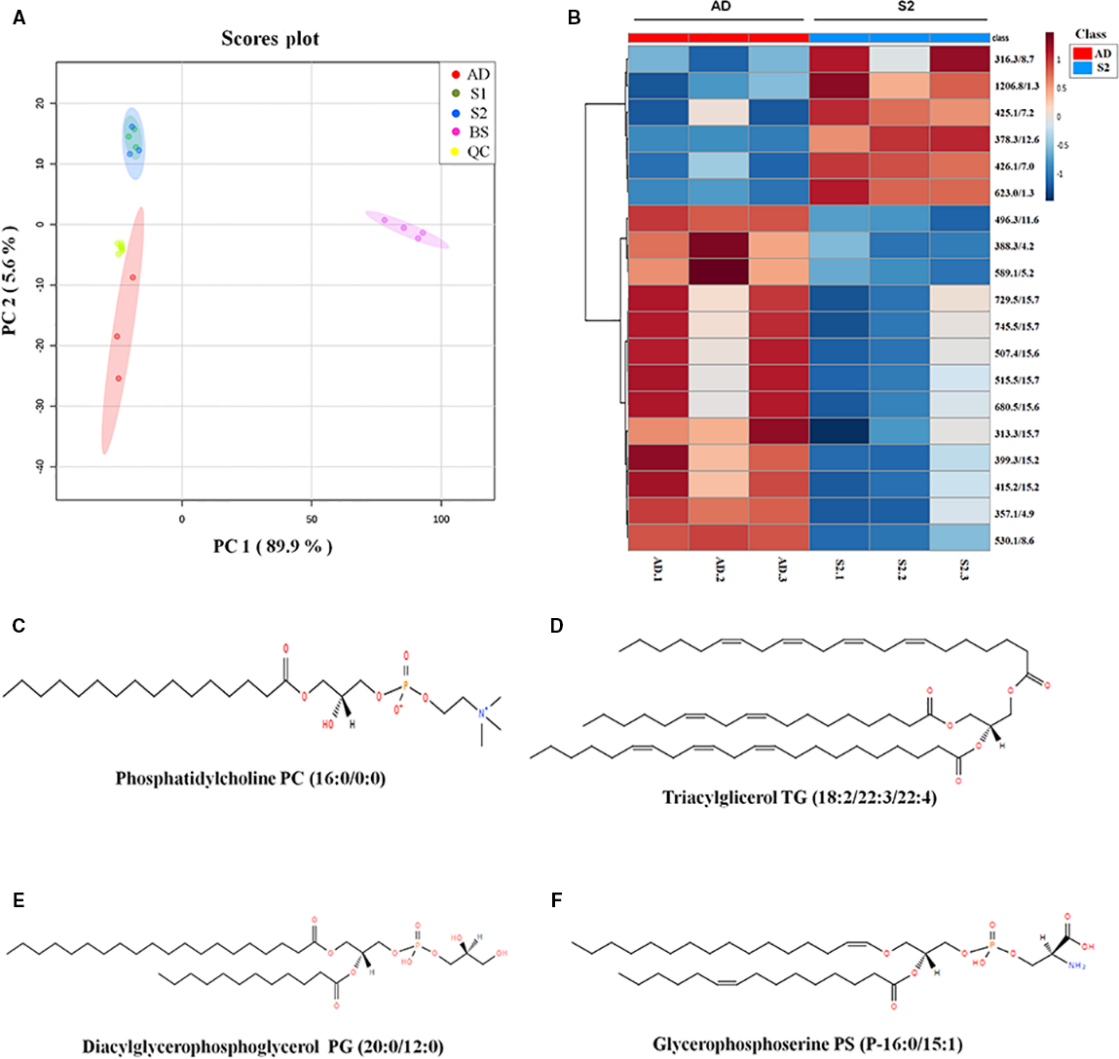
Metabolomic analysis of exosomes derived from Mel1 patient‐derived cell line. (A) PCA score plots based on HPLC/MS data of exosome samples derived from adherent cells (red), primary melanospheres (green), secondary melanospheres (blue), QC samples (yellow) and BS samples (pink). (B) Heat map showing the significantly different metabolites when comparing exosomes derived from adherent cells (red) and secondary melanospheres (blue). Each row on the heat map represent a unique metabolite with a characteristic mass‐to‐charge ratio and retention time, while each column represents one exosome sample. The colour code represents the normalized intensity with which each metabolite is detected. Blue represents a decreasing trend, while red represents a rising trend. (C) Chemical structure of candidate biomarker 1‐hexadecanoyl‐sn‐glycero‐3‐phosphocholine PC (16:0/0:0). (D) Chemical structure of candidate biomarker triacylglycerol TG (18:2/22:3/22:4). (E) Chemical structure of candidate biomarker diacylglycerophosphoglycerol PG (20:0/12:0). (F) Chemical structure of candidate biomarker glycerophosphoserine PS (P‐16:0/15:1).

After this filtering process, 138 candidates (5.5% of total mass signals) were considered and selected as differentially expressed in exosome samples versus mobile‐phase solvent samples. Next, ANOVA (*P*‐value < 0.05) and fold change (FC) > 1.5 filtering were performed for multiple comparison on the three groups of samples (adherent cells, primary spheres and secondary spheres), and *post hoc* analysis using Tukey's honestly significant difference (Tukey's HSD) was applied to identify significant metabolite changes. As an outcome, 19 differential *m/z* signals met these criteria (Table S1). After the selection of those differential *m/z* signals, samples were analysed by principal component analysis (PCA) and partial least squares discriminant analysis (PLS‐DA). The PCA score plot for all the analysed sample groups is shown in Fig. 4A. The clustering or spreading of quality control (QC) samples allows assessing the quality of the analytical system performance. Blank solvent (BS) samples were clearly separated from biological samples (Fig. 4A). Exosome samples derived from adherent cells, and primary and secondary spheres were clearly separated from each other along the first principal component (PC1) and the second principal component (PC2), which describe about 89.9% and 5.6%, respectively, of the total data variability remaining after filtering. The group separation observed in the PCA scores plot indicated a differential pattern of the metabolites found in exosomes isolated from adherent cells compared with primary and secondary spheres (Fig. 4A).

The PCA score plot for the three‐group comparison between exosomes derived from adherent cells, and primary and secondary CSCs is shown in Fig. S2A, while the corresponding heat map showing the differential abundance of those metabolites found as statistically different in those three comparison groups is shown in Fig. S2B.

However, as can be clearly seen in the PCA score plot, the greatest metabolic differences were found between adhered cells and CSC melanospheres, but not between primary and secondary spheres. For that reason, a filtering by *t*‐test (*P*‐value < 0.05) and FC (FC > 1.5) was also performed comparing adhered cells with secondary sphere samples, as they were those that possessed more stemness properties (Fig. 1). When comparing these two groups, 19 differential *m/z* signals also met these criteria (Table 1). Based on PLS‐DA models, exosome samples derived from adherent cells and secondary spheres were discriminated with a *R* ^2^ of 0.99 and a *Q* ^2^ of 0.97, exceeding the threshold values accepted in metabolomic experiments (*R* ^2^ > 0.7 and *Q* ^2^ > 0.4) [33]. Accordingly, the heat map displayed a clear differential pattern of metabolite expression across samples of exosomes derived from both Mel1 adherent cells and Mel1 secondary spheres (Fig. 4B).

**Table 1:** Differential metabolites found in the two‐group comparison between exosomes derived from adherent cells (ADs) and secondary spheres (S2), in Mel1 patient‐derived cell line exosomes. *m/z*, mass‐to‐charge ratio; RT, retention time (min).

AD/S2^a^			
*m/z*	RT	*P*‐value^b^	FC^c^
313.2714	15.7	0.0318	1.7911 (↑)
316.3215	8.7	0.03372	0.6633 (↓)
357.1404	4.9	0.00163	11.1627 (↑)
378.3214	12.6	0.0008	0.3929 (↓)
388.2521	4.2	0.00685	1.5919 (↑)
399.2608	15.2	0.01546	1.8870 (↑)
415.2359	15.2	0.01587	1.8891 (↑)
425.136	7.2	0.01171	0.1966 (↓)
426.1372	7.0	0.00049	0.1124 (↓)
496.3409	11.6	6.48 × 10^−5^	4.1367 (↑)
507.4077	15.6	0.04807	3.1055 (↑)
515.3962	15.7	0.04258	2.2071 (↑)
530.1469	8.6	2.24 × 10^−5^	18.1682 (↑)
589.1461	5.2	0.04382	10.2656 (↑)
623.0031	1.3	0.00377	0.0106 (↓)
680.5277	15.6	0.04514	2.0574 (↑)
729.5293	15.7	0.04517	2.2063 (↑)
745.5143	15.7	0.04205	2.2513 (↑)
1206.827	1.3	0.009	0.4205 (↓)

^a^ Two‐group comparison (*t*‐test).

^b^ *P*‐value corresponding to univariate statistical analysis (*t*‐test). Only peaks with a *P*‐value < 0.05 were selected.

^c^ Fold change expressed as the ratio of the two averages (AD/S2). Only peaks with a fold change > 1.5 or < 0.66 were selected. The arrows indicate whether the metabolite is increased (↑) or decreased (↓) in AD relative to S2.

### 3.4 Structural identification of selected differential metabolites in primary patient Mel1 cells

Metabolite identification was carried out according to the criteria explained in Materials and Methods section. In addition, we established two identification levels: (1) molecular formula matched with isotopic profile and compound databases and (2) experimental fragmentation spectrum matched in spectral databases. As a result, it was possible to assign the following tentative identifications for differential peaks between exosome samples derived from adherent cells and those derived from CSC melanospheres (Table 2): *m/z* 496.3381 corresponds to the glycerophosphocholine PC 16:0/0:0 (http://www.lipidmaps.org/data/LMSDRecord.php?LMID=LMGP01050018) (Fig. 4C); *m/z* 515.3962 corresponds to the triacylglycerol TG (18:2/22:3/22:4) (http://www.lipidmaps.org/data/LMSDRecord.php?LMID=LMGL03012177) (Fig. 4D); *m/z* 729.5293 corresponds to the diacylglycerophosphoglycerol PG (20:0/12:0) (http://www.lipidmaps.org/data/LMSDRecord.php?LMID=LMGP04010867) (Fig. 4E); and *m/z* 745.5143 corresponds to the glycerophosphoserine PS (P‐16:0/15:1) (http://www.lipidmaps.org/data/LMSDRecord.php?LMID=LMGP03030006) (Fig. 4F).

**Table 2:** Differential metabolites tentatively identified in Mel1 patient‐derived cell line exosomes based on MS/MS fragmentation spectra and database search. *m/z*, mass‐to‐charge ratio; RT, retention time (min).

*m/z*	RT	Tentative ID^a^	Molecular formula^b^	Mass error^c^	*P*‐value^d^	FC^e^	IL^f^
496.3381	11.61	LPC (16:0)	C_24_H_50_NO_7_P	3	6.48 × 10^−5^	4.1367 (↑)	2
515.3962	15.72	TG(18:2/22:3/22:4)	C_65_H_108_O_6_	0	0.04258	2.2071 (↑)	2
729.5293	15.71	PG(20:0/12:0)	C_38_H_75_O_10_P	4	0.04517	2.2063 (↑)	2
745.5143	15.70	PS(P‐16:0/15:1)	C_37_H_70_NO_9_P	2	0.04205	2.2513 (↑)	2

^a^ Common name of the tentatively identified metabolite according to MS/MS fragmentation spectra and database search.

^b^ Molecular formula of the tentatively identified metabolite.

^c^ Mass error (p.p.m.).

^d^ *P*‐value corresponding to univariate statistical analyses (*t*‐test). Only peaks with a *P*‐value < 0.05 were selected.

^e^ Fold change expressed as the ratio of the two averages (AD/S2). Only peaks with a fold change > 1.5 or < 0.66 were selected. The arrows indicate whether the metabolite is increased (↑) or decreased (↓) in AD relative to S2.

^f^ Identification level: (1) molecular formula matched in compound databases and (2) experimental fragmentation spectrum matched in spectral databases.

We were not able to identify the rest of *m/z* signals or assign them a biologically coherent molecular formula, according to the same mentioned criteria and the identification rules described by Kind and Fiehn [35].

The four tentatively identified and other nine unidentified ions have significant differences between the groups, being more abundant in exosome samples derived from adherent cells compared with those from secondary spheres. However, other six unidentified signals were more abundant in CSC Mel1‐derived exosomes compared with those from adherent cells (Fig. 4B).

### 3.5 LC–HRMS metabolomic analysis of exosomes derived from serum of patients with MM

**Fig. 5:**
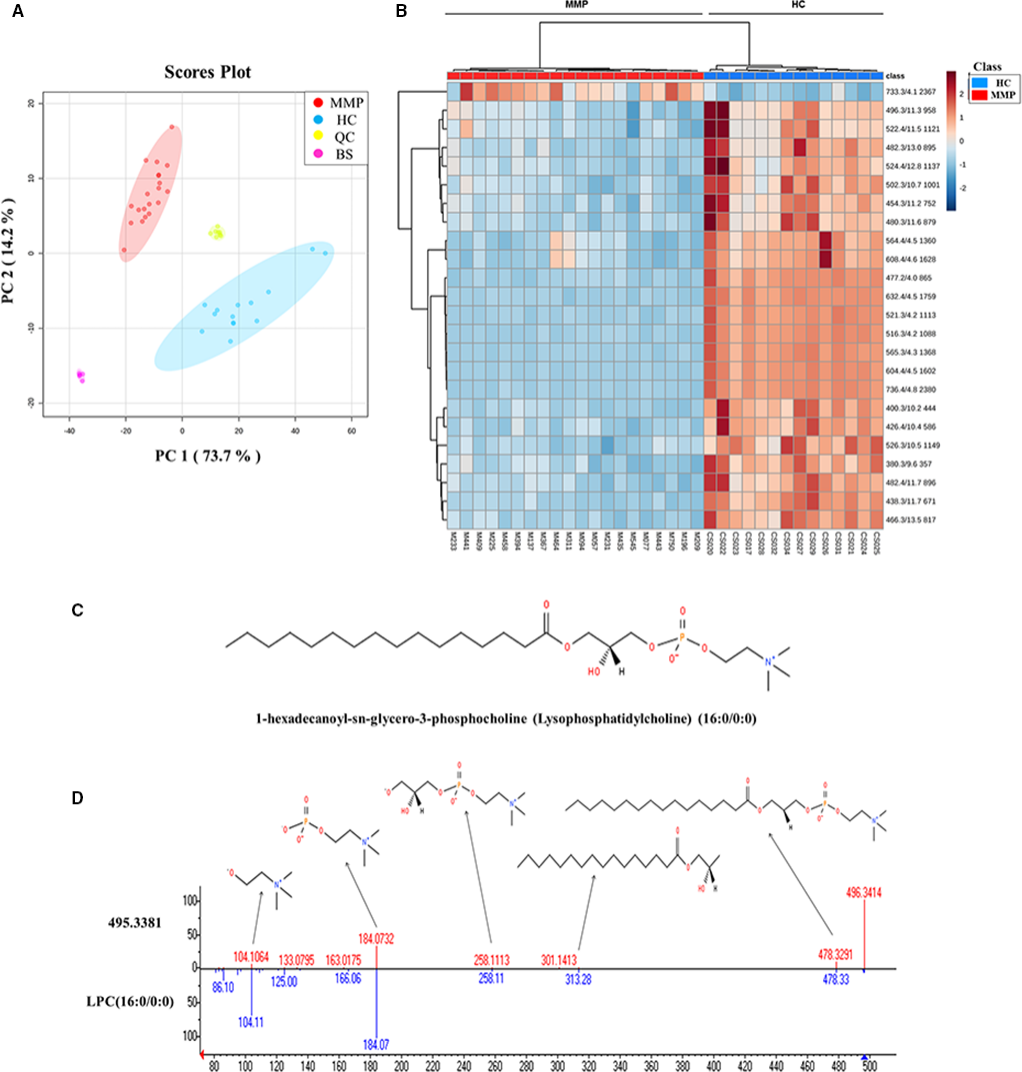
Metabolomic analysis of serum‐derived exosomes from both patients with malignant melanoma and healthy individuals. (A) PCA score plots based on HPLC/MS data of serum‐derived exosome samples from MMPs (red), HCs (blue), QC samples (yellow) and BS samples (pink). (B) Heat map showing the changing intensity patterns of significantly different metabolites of two‐group comparison: exosome samples derived from MMPs (red) versus exosomes derived from HCs (blue). (C) Chemical structure of candidate biomarker 1‐hexadecanoyl‐sn‐glycero‐3‐phosphocholine PC (16:0/0:0); (D) representative fragmentation spectrum of candidate biomarker PC (16:0/0:0). Within the product ion spectra arising from the [M + H]^+^ ions of this molecule, different specific fragments were found, such as the *m/z* 184, 104, 258, 321 or 478 ions, corresponding to characteristic molecule fragments.

Analogously to Mel1‐derived exosomes, we carried out a metabolomic analysis in the same experimental conditions with the aim of exploring metabolomic differences between serum‐derived exosomes from MMPs at different stages of the disease and HCs. As an outcome, 93 differential (*t*‐test *P*‐value < 0.05 and FC < 1.5) *m/z* signals were found. These signals were analysed by PCA and PLS‐DA, obtaining a clear separation of exosome samples derived from MMP and HC serum within the PCA score plot along PC1 and PC2 (Fig. 5A), which describe about 73.7% and 14.2%, respectively, of the total data variability. In PLS‐DA models, exosome samples derived from MMP and HC serum were discriminated with an *R* ^2^ of 0.99 and *Q* ^2^ of 0.98.

Due to the greater complexity of the patient‐derived serum composition, we found a larger number of differentially expressed metabolites compared with Mel1 primary cell line. Since metabolite identification is the most laborious and time‐consuming task in the metabolomic workflow, we applied the variable importance in projection (VIP) technique as an additional independent variable selection method in order to achieve a more affordable set of metabolites in terms of identification [36]. Following the greater‐than‐one rule, which is usually considered for detecting variables with the greatest importance in the projection [36], 24 differentially expressed metabolites were selected (Table 3). The heat map representing the differential abundance of these selected metabolites between exosome samples derived from MMPs and HCs is shown in Fig. 5B. As can be observed, 23 metabolites were more abundant in exosome samples derived from HCs, compared with those from MMPs, and only 1 metabolite (not identified) was higher in MMPs in comparison with HCs (Fig. 5B, Table 3). Interestingly, the metabolite with *m/z* 496.3381, identified as 1‐hexadecanoyl‐sn‐glycero‐3‐phosphocholine (PC 16:0/0:0), was found to be overexpressed in exosomes derived from both HC serum and adherent Mel1 cells, compared with MMP serum and CSC Mel1 cells, respectively.

**Table 3:** Potential biomarkers differentially expressed in MMP compared with HC serum‐derived exosomes based on MS/MS fragmentation spectra and database search. *m/z*, mass‐to‐charge ratio; RT, retention time (min).

*m/z*	RT	Tentative ID^a^	Molecular formula^b^	Mass error^c^	*P*‐value^d^	FC^e^	AUC^f^	VIP^g^	IL^h^
380.2551	9.64	Sphingosine‐1‐phosphate	C_18_H_38_NO_5_P	2	1.31 × 10^−13^	3.57 (↑)	1	1.03	2
400.3419	10.19	Palmitoylcarnitine	C_23_H_45_NO_4_	1	9.71 × 10^−16^	6.07 (↑)	1	1.26	2
426.3570	10.41	Elaidic carnitine	C_25_H_47_NO_4_	2	2.54 × 10^−14^	3.44 (↑)	1	1.13	2
438.2987	11.69	PE(P‐16:0/0:0	C_21_H_44_NO_6_P	2	4.74 × 10^−19^	5.18 (↑)	1	1.33	2
454.2902	11.20	PE(16:0/0:0)	C_21_H_44_NO_7_P	6	1.99 × 10^−09^	3.72 (↑)	1	1.23	2
466.3302	13.55	Glycerophospholipid‐related compound	C_23_H_48_NO_6_P	2	1.16 × 10^−15^	3.31 (↑)	1	1.18	1
477.2309	3.99	–	–	–	2.33 × 10^−24^	9.84 (↑)	1	1.36	–
480.3080	11.64	PE(18:1/0:0)	C_23_H_46_NO_7_P	1	2.11 × 10^−09^	3.33 (↑)	1	1.23	2
482.3251	12.95	PE(18:0/0:0)	C_23_H_48_NO_7_P	2	5.19 × 10^−10^	3.71 (↑)	1	1.45	2
482.3585	11.71	PC(O‐16:0/0:0)	C_24_H_52_NO_6_P	4	1.11 × 10^−12^	2.54 (↑)	1	1.1	2
496.3391	11.28	LPC (16:0)	C_24_H_50_NO_7_P	1	1.18 × 10^−06^	3.89 (↑)	0.99	2.28	2
502.2917	10.68	PE(20:4/0:0)	C_25_H_44_NO_7_P	2	1.24 × 10^−10^	3.25 (↑)	0.99	1.15	2
516.3009	4.17	Taurallocholic acid	C_26_H_45_NO_7_S	4	7.39 × 10^−28^	22.23 (↑)	1	1.72	1
521.2544	4.17	–	–	–	3.90 × 10^−27^	22.86 (↑)	1	1.6	–
522.3551	11.47	PC(18:1/0:0)	C_26_H_52_NO_7_P	1	3.83 × 10^−06^	2.40 (↑)	0.96	1	2
524.3698	12.76	PC(18:0/0:0)	C_26_H_54_NO_7_P	2	3.95 × 10^−08^	4.00 (↑)	0.98	1.57	2
526.2902	10.47	PE(22:6/0:0)	C_27_H_44_NO_7_P	5	2.60 × 10^−13^	3.82 (↑)	1	1.06	2
564.3588	4.49	Ganglioside GM3 (d18:0/14:0)	C_55_H_102_N_2_O_21_	5	5.80 × 10^−12^	2.19 (↑)	0.99	1.07	1
565.2809	4.32	–	–	–	8.84 × 10^−28^	13.19 (↑)	1	1.58	–
604.3544	4.46	Presqualene diphosphate	C_30_H_52_O_7_P_2_	0.6	4.46 × 10^−29^	24.62 (↑)	1	1.99	1
608.3849	4.61	–	–	–	1.37 × 10^−11^	2.33 (↑)	0.99	1.08	–
632.3827	4.46	–	–	–	1.20 × 10^−28^	44.92 (↑)	1	1.58	–
733.3346	4.15	–	–	–	1.08 × 10^−07^	0.13 (↓)	0.95	1.02	–
736.4321	4.76	Glycerophospholipid‐related compound	C_38_H_68_NO_8_P	1	4.47 × 10^−29^	52.18 (↑)	1	1.97	1

^a^ Common name of the tentatively identified metabolite according to MS/MS fragmentation spectra and database search.

^b^ Molecular formula of the tentatively identified metabolite.

^c^ Mass error (p.p.m.).

^d^ *P*‐value corresponding to univariate statistical analyses (*t*‐test). Only peaks with a *P*‐value < 0.05 were selected.

^e^ Fold change expressed as the ratio of the two averages (HC/MMP). Only peaks with a fold change > 1.5 or < 0.66 were selected. The arrows indicate whether the metabolite is increased (↑) or decreased (↓) in HC relative to MMP.

^f^ Area under the curve corresponding to ROC curve analyses.

^g^ VIP value corresponding to variable importance in the projection selection technique.

^h^ Identification level: (1) molecular formula matched in compound databases and (2) experimental fragmentation spectrum matched in spectral databases.

Additionally, the area under the curve (AUC) values were calculated from receiver operating characteristic (ROC) curve analysis in order to assess the potential clinical utility of the previously selected metabolites, displaying all values close or equal to 1, which suggest they could be considered as potential diagnostic biomarkers. Some of those selected differential peaks between the exosome samples derived from MMPs and those derived from HCs were tentatively identified (Table 3). Following the same criteria previously described, we established two identification levels. Figure 5C,D shows the chemical structure and the corresponding interpretation of fragmentation spectrum of *m/z* 496.3381 identified as PC 16:0/0:0, as an example of one of those tentatively identified metabolites. For some of the *m/z* signals, a tentative identification was not possible following the same criteria [35]. However, they could also be considered as potential biomarkers, since all of them showed high AUC values.

## 4 Discussion

Metastatic melanoma is the most aggressive and life‐threatening form of skin cancer. Despite advances in its treatment, the incidence rate and mortality have continued increasing worldwide over the past few decades. Therefore, there is an urgent need for further understanding of the underlying mechanisms of this neoplasia, and for discovering clinically useful biomarkers. The diagnosis of MM currently remains challenging and the procedure is still invasive, expensive and time‐consuming, since it requires the removal and analysis of the primary melanoma, detection of high‐risk markers and sentinel lymph node biopsy to determine the presence and stage of metastatic disease [37]. There are several promising tissue and serological biomarkers for predicting melanoma progression and overall patient survival [38]. Among the serological biomarkers, only S100 calcium‐binding protein B (S100B) and lactate dehydrogenase (LDH) have some value for predicting progression to an advanced stage of the disease but do not translate into adequate therapeutic intervention and survival [39]. However, there are currently no reliable serum‐derived biomarkers for early detection of the disease or prognostic markers for early‐stage melanoma patients. This underscores the need to identify reliable noninvasive blood‐based biomarkers that allow an economical, rapid and standardized detection of MM in its earliest stages, the identification of patients at the highest risk of metastatic recurrence and the prediction of their treatment response in order to improve patient outcome.

In this context, exosomes represent a novel source of noninvasive biomarkers since they and their cargo can be easily isolated from most body fluids using minimally invasive techniques [40]. They are considered as a subtype of sEVs, which may comprise exosomes and other small‐sized vesicles sharing densities and markers [7]. To date, it is known that the composition of sEVs is not a random sample of cell content, but rather is assembled by a highly selective process whose nature remains unclear [41]. Moreover, while in some instances the antigens found on the surface of microvesicles (e.g. lineage markers) could resemble those of their producing cells [42], several studies suggest that, especially, exosomes contain a more unique protein and RNA cargo. Thus, exosomes tend to be enriched in glycoproteins compared with the secreting cells [43]. In addition, sEVs imperatively comprise a lipid moiety, and their phosphatidylserine, cholesterol, sphingomyelin and glycosphingolipid content is richer than their cellular sources [44, 45]. Thus, the studies that originally reported their presence in blood determined that the sEV membrane could support the coagulation cascade by exposition on their surface of phosphatidylserine [46]. Furthermore, sEVs also interact with secreted phospholipases to generate eicosanoids, which regulate the transfer of cargo into a cellular recipient. Eicosanoids, potent bioactive lipid mediators, are useful as biomarkers and contribute to a variety of biological functions, including modulation of distal immune responses, angiogenesis and tumour progression in cancer context [47]. The role of the various lipidic pathways is crucial in the biogenesis and functions of microvesicles and exosomes. For instance, tumour‐derived exosomes enriched in prostaglandins and free fatty acids (including arachidonic acid) participate in the formation of a favourable microenvironment for tumour growth [48]. In this context, sEVs contribute with their lipid molecules or their lipid‐related enzymes to several pathophysiologies, playing an important role in cancer [49].

Exosomes are actively secreted by cancer cells at a higher rate than by normal cells. In particular, melanoma cells seem to produce a large quantity of these microvesicles [50], in contrast to normal melanocytes [51]. Moreover, a melanoma‐specific exosomal signature, which correlates with tumour burden and metastasis, was identified in blood from patients with MM at stage IV [14]. Malignant melanoma is characterized by an extraordinary heterogeneity, propensity for dissemination to distant organs and resistance to chemotherapy, which results from the unique characteristics of melanoma CSCs [52].

In this study, we have set up a broadly applicable approach for metabolomic profiling of exosomes isolated from cell culture media of Mel1 melanoma CSCs and from serum of patients with MM at different stages in order to identify potential clinically useful biomarkers with prognostic/diagnostic value. First, we confirmed that the primary Mel1 cell line obtained from a metastatic MM growing in serum‐free and anchorage‐independent conditions displayed both CSC‐like functional and phenotypic properties. Mel1 cells growing as spheres possess self‐renewal ability and clonogenicity. The spheres were enriched in cells with high ALDH activity, overexpressing CD20 and CD44 surface markers and presenting a great SP rate. All these properties have been described as characteristics of CSCs [29]. The isolation of exosomes from cell culture supernatant was performed by ultracentrifugation and confirmed by TEM, AFM and western blot assays. Next, we characterized their size by NanoSight showing particles with diameters of around 100 nm, confirming the specific enrichment in exosomes [14, 17]. Mel1 exosomes were positive for CD9, CD63 and Alix exosomal markers, and also for CD271 melanoma CSC marker. Previous studies on patients with melanoma have shown that CD271+ is a good candidate marker to unequivocally identify CSC subpopulation [53]. However, it is important to highlight that, based on the exosome isolation and characterization protocols carried out in this study, the term ‘exosomes’ applied in this work actually refers to sEVs, a general term proposed in the ‘MISEV’ guidelines [7], comprising exosomes but also other vesicles which share size, density and markers.

The metabolomic profile of exosomes analysed through MS revealed significant differences in the metabolomic fingerprint of exosomes derived from CSCs as compared to tumour adherent (more differentiated) cells in Mel1 MM primary cell line and also in the exosomes derived from MMPs at different stages compared with HCs. ROC curves are frequently used in biomedical informatics research to evaluate classification and prediction models for decision support, diagnosis and prognosis. Thus, it is possible within a metabolomic study to calculate ROC curves for each potential biomarker in order to assess its potential clinical utility in terms of AUC [34]. In this regard, we calculated the AUC for each selected candidate biomarkers in patients' serum‐derived exosomes, and we obtained values close or equal to 1. Considering that AUC values over 0.8 indicate a good predictor model, our results (Tables 1 and 3) suggest that these metabolites could be used as a panel of clinically useful biomarkers.

Metabolic reprogramming is firmly established as a hallmark of cancer [54], and lipids have been described to exert multiple biochemical functions during cancer development. Several lipids, including sterols, di‐/triacylglycerols and phospholipids, are integral part of biological membranes and are also used for energy storage, production and cellular signalling. Fatty acids (FAs) are indispensable for lipid biosynthesis. Disruption of lipid metabolism, especially FA synthesis (FAS) and fatty acid oxidation (FAO), has become increasingly recognized as an important metabolic rewiring phenomenon in tumour cells [55]. Glycolipids and phospholipids (phosphatidylcholine and phosphatidylethanolamine) along with cholesterol are major components of biological membranes and markedly influence membrane fluidity [55]. In addition to their structural roles, lipids also orchestrate signal transduction cascades and can also be broken down into bioactive lipid mediators, which regulate several carcinogenic processes, such as cell growth, cell migration and metastasis [56].

In our study, we found a decreased expression between exosome samples derived from CSC melanospheres and those derived from adherent‐differentiated tumour cells of four tentatively identified metabolites from different lipid classes, such as glycerophosphoglycerols [PG(20:0/12:0)], glycerophosphoserines [PS(P‐16:0/15:1)], triacylglycerols [TG(18:2/22:3/22:4)] and glycerophosphocholines (PC 16:0/0:0). Interestingly, we found that PC 16:0/0:0 expression was reduced in both Mel1 CSCs and MMPs in comparison with Mel1 differentiated tumour cells and HCs, respectively. In line with these results, previous studies reported the reduced expression of PC 16:0/0:0 associated with malignant diseases, such as colorectal cancer [57], digestive tract tumours or renal cell carcinoma [58]. Moreover, higher levels of other glycerophospholipids such as LysoPC 18:0/0:0 have been consistently related to lower risks of breast, prostate and colorectal cancer [59]. Other studies have shown that the serological lipidomic profile of prostate cancer patients revealed several putative lipids that might serve as diagnostic biomarkers of this neoplasm [60].

The metastatic potential of cancer cells correlates with the expression of genes involved in fatty acid synthesis, oxidation and intracellular lipid storage. It has been shown that enzymes involved in lipid metabolism play a role in metastasis. For example, stearoyl‐CoA desaturase (SCD) and long‐chain fatty acyl synthetase (ACSL) 1 and 4 cooperate to induce epithelial‐to‐mesenchymal transition resulting in an increased invasion potential of colon cancer cells [61]. Furthermore, it has been suggested that the rapid extracellular hydrolysis of phospholipids like PC 16:0/0:0 by metastatic tumour cells and the subsequent cellular uptake of the resulting free fatty acids (FFAs) seems to be a necessary prerequisite for metastatic potential of epithelial tumour cells, probably for generating pro‐metastatic lipid second messengers [62].

In this work, we also detected significant differences in other metabolites in the serum‐derived exosomes from HCs compared with MMPs. For example, we found lower levels of the lysophospholipid sphingosine 1‐phosphate (S1P) in serum‐derived exosomes from MMPs than in HCs. This is consistent with previous studies that had suggested this molecule as a potential serum biomarker for hepatocellular carcinoma (HCC) diagnosis, which was also found to be lower in HCC patients [63]. Other differential metabolites that were lower in MMP serum‐derived exosomes were palmitoylcarnitine and elaidic carnitine. Recently, the role of the carnitine system has been described in the metabolic plasticity phenomenon, a mechanism through which cancer cells are able to become more aggressive and metastasize [64]. In agreement with our results, a similar previous study, aimed at characterizing the metabolomic serum profile of HCC patients in a Korean prospective cohort, also showed lower levels of palmitoylcarnitine in HCC patients, compared with healthy individuals [65]. Another metabolomic study reported the potential use of this metabolite, among others, as a predictive serum biomarker in non‐small cell lung cancer [66]. We also found lower levels of several phospholipid‐related compounds such as phosphatidylcholines (PCs) and phosphatidylethanolamines (PEs) in MMP exosomal extracts versus HC extracts (see Table 3). Changes in specific phospholipid (PL) levels in tissues, cells and body fluids such as urine, plasma or serum have clearly been demonstrated to be associated with cancer [67]. Yang *et al*. suggested that specific differential PLs found in the plasma of breast cancer patients could be useful for diagnosis purposes [68], and Waki *et al*. also reported PL differences in breast CSCs and non‐stem cancer cells (NSCCs) [69]. In colorectal cancer, decreased LPC levels in serum patients have potential for use as diagnosis biomarkers [70], since lower LPC levels could be associated with the loss of body weight and inflammation, but could also indicate a higher LPC decomposition rate to support cancer metabolism. Another study showed that some PL species, including LPC (16:0), LPC (18:0), PC (16:0) and PC (18:0), were significantly less present in HCC and LC (liver cirrhosis) patients, compared with HCs [71]. Another tentatively identified metabolite, also found at lower levels in serum‐derived exosomes from MMPs, is the glycosphingolipid ganglioside GM3 (d18:1/16:00), a component of cell plasma membrane that modulates cell signal transduction events. It has been reported that GM3 downregulates the invasiveness capacity of human bladder cancer cells and also that exogenously added GM3 can prevent haptotactic cell migration in colorectal cancer cell lines [72]. For the rest of metabolites, it was not possible to give an accurate mass or MS/MS spectra‐based putative identifications using several databases. This still represents a major challenge in the field of metabolomics. However, it could be clinically useful to explore exosome‐associated metabolomic *m/z* signatures related to several stages of MM. All these results found in both patients and CSCs suggest the importance of structural lipids detected in exosomes of patients with MM as biomarkers in early detection of patients with a high risk of MM and their potential in the determination of aggressiveness or therapeutic monitoring. However, since these results have been obtained from a limited number of patients, further studies with higher number of MMPs are necessary.

## 5 Conclusions

In conclusion, to our knowledge, this is the first metabolomic‐based study aimed at characterizing the cargo and composition of exosomes secreted by melanoma CSCs, in order to identify diagnosis and prognosis biomarkers of MM progression. Our results provide evidence for using MS to detect exosome lipid metabolites as a rich source of biomarkers' discovery for use in translational research and precision medicine in oncology.

## Conflict of interest

The authors declare no conflict of interest.

## Author contributions

JLP‐F and MBG‐O designed and performed experiments, data analysis, result interpretation and preparation of the manuscript; MG‐G and JPdP contributed to the design, data analysis, result interpretation and preparation of the manuscript; MBG‐O, JV, JMJ, FMA‐F, SA‐S and VA recruited MMP and HC serum samples; MAG, CD, HB, HP and FV contributed to the results interpretation and preparation of the manuscript; and JAM conceived the study, designed experiments, prepared the manuscript and got the funding.

## Supporting information


**Fig. S1.** Characterization of exosomes derived from Mel1 differentiated tumour cells.
**Fig. S2.** Metabolomic analysis of exosomes derived from Mel1 patient‐derived cell line.
**Table S1.** Significantly different metabolites found in the three‐group comparison between adherent cells, primary spheres and secondary spheres, in Mel1 patient‐derived cell line exosomes.
