# Supplementary material for: Metabolomic profile of cancer stem cell‐derived exosomes from patients with malignant melanoma
Source: Mol Oncol. 2020 Nov 25;15(2):407–28. doi: 10.1002/1878-0261.12823 (PMC7858120; doi:10.1002/1878-0261.12823)
Supplement: Supplementary file 1 — Fig. S1. Characterization of exosomes derived from Mel1 differentiated tumour cells. Fig. S2. Metabolomic analysis of exosomes derived from Mel1 patient‐derived cell line. Table S1. Significantly different metabolites found in the three‐group comparison between adherent cells, primary spheres and secondary spheres, in Mel1 patient‐derived cell line exosomes. [file MOL2-15-407-s001.doc]

**Supplementary Figures**


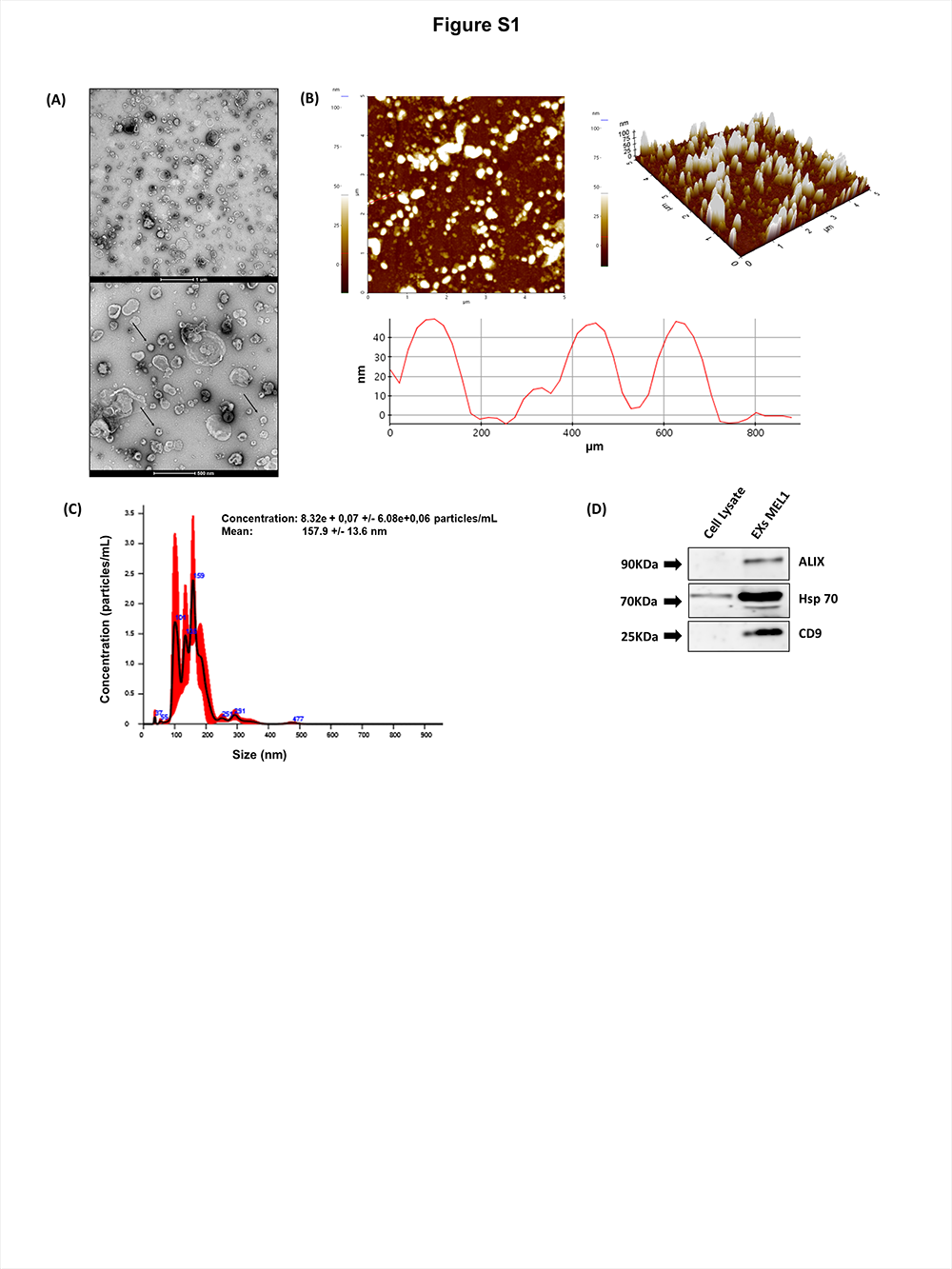


**Figure S1. Characterization of exosomes derived from Mel1 differentiated tumour cells**. **(A)** Transmission electron microscopy images of isolated exosomes with a saucer-like shape limited by a lipid bilayer. EVs isolated from Mel1 culture supernatants had diameters ranging from ~50–240 nm. Black arrow heads point to exosomes; **(B)** Topography of exosomes derived from Mel1 adherent cells observed under atomic force microscopy (AFM). Exosomes on a mica surface revealed heterogeneity in size and shape as well as forming aggregates in both 2-dimensional 2D (left) images and 3D profiles (right). Acquisition areas were 5 x 5 µm^2^; **(C)** The size distribution of exosomes isolated from Mel1 adherent cells was analyzed by NTA; **(D)** Western blot analysis of CD9, Alix and the Hsp70 exosomal surface markers.


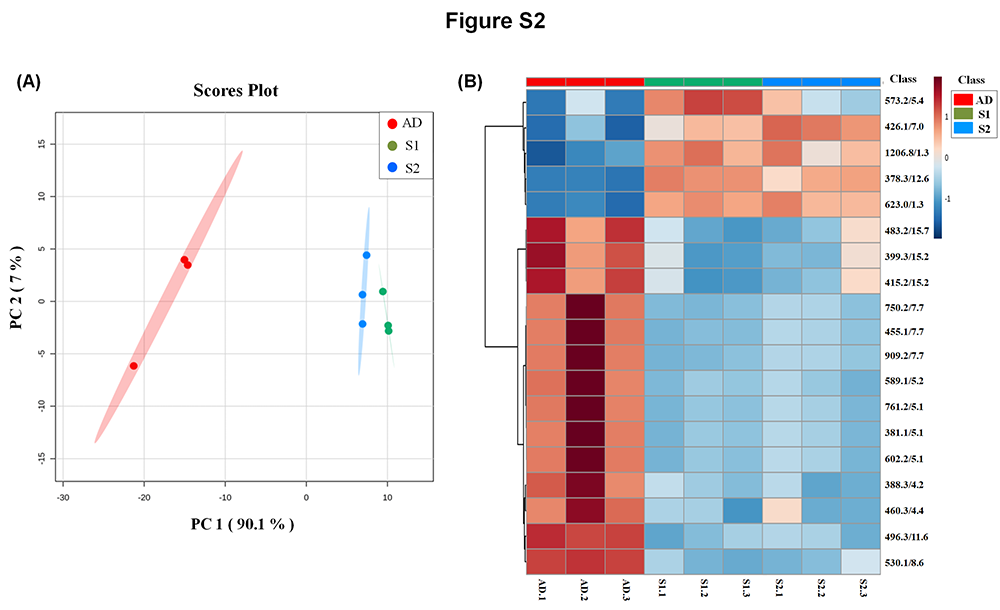


**Figure S2**. **Metabolomic analysis of exosomes derived from Mel1 patient-derived cell line.** **(A)** PCA scores plots based on HPLC/MS data of exosome samples derived from adherent cells (red), primary spheres (green) and secondary spheres (blue). **(B)** Heatmap showing the significantly different metabolites when comparing exosomes derived from adherent cells (red), primary spheres (green) and secondary spheres (blue). Each row on the heatmap represents a unique metabolite with a characteristic mass-to-charge ratio and retention time while each column represents one exosome sample. The colour code (blue to red) represents the normalized intensity with which each metabolite is detected.

**Supplementary tables**

**Table S1.** Significantly different metabolites found in the three-group comparison between adherent cells (AD), primary spheres (S1) and secondary spheres (S2), in Mel1 patient-derived cell line exosomes.

| AD/S1/S2 ^1^ | | |
| --- | --- | --- |
| m/z ^2^ | RT ^3^ | P-value ^4^ |
| 378.3214 | 12.6 | 7.62x10^-6^ |
| 381.0997 | 5.1 | 0.001326 |
| 388.2521 | 4.2 | 0.0011 |
| 399.2608 | 15.2 | 0.00637 |
| 415.2359 | 15.2 | 0.006676 |
| 426.1372 | 7.0 | 0.000571 |
| 455.1165 | 7.7 | 0.000862 |
| 460.3096 | 4.4 | 0.005243 |
| 483.2172 | 15.7 | 0.00584 |
| 496.3409 | 11.6 | 1.91x10^-5^ |
| 530.1469 | 8.6 | 5.87x10^-5^ |
| 573.2039 | 5.4 | 0.006179 |
| 589.1461 | 5.2 | 0.001116 |
| 602.1573 | 5.1 | 0.001039 |
| 623.0031 | 1.3 | 8.64x10^-6^ |
| 750.1882 | 7.7 | 0.000799 |
| 761.1923 | 5.1 | 0.00124 |
| 909.2258 | 7.7 | 0.000875 |
| 1206.827 | 1.3 | 0.000903 |

^1^ Three-group comparison (ANOVA); ^2^ Mass-to-charge ratio; ^3^ Retention time (min); ^4^ p-value corresponding to univariate statistical analysis (ANOVA). Only peaks with a p-value < 0.05 were selected.
